# Food to Prevent Vascular Calcification in Chronic Kidney Disease

**DOI:** 10.3390/nu16050617

**Published:** 2024-02-23

**Authors:** Diana Moldovan, Crina Rusu, Alina Potra, Dacian Tirinescu, Maria Ticala, Ina Kacso

**Affiliations:** 1 Department of Nephrology, “Iuliu Hatieganu” University of Medicine and Pharmacy Cluj-Napoca, 400347 Cluj-Napoca, Romania; 2 Nephrology Clinic, Emergency County Hospital Cluj-Napoca, 400347 Cluj-Napoca, Romania

**Keywords:** food, nutrition, lifestyle, vascular calcification, chronic kidney disease

## Abstract

Vascular calcification (VC) is a consequence of chronic kidney disease (CKD) which is of paramount importance regarding the survival of CKD patients. VC is far from being controlled with actual medication; as a result, in recent years, diet modulation has become more compelling. The concept of medical nutritional therapy points out the idea that food may prevent or treat diseases. The aim of this review was to evaluate the influence of food habits and nutritional intervention in the occurrence and progression of VC in CKD. Evidence reports the harmfulness of ultra-processed food, food additives, and animal-based proteins due to the increased intake of high absorbable phosphorus, the scarcity of fibers, and the increased production of uremic toxins. Available data are more supportive of a plant-dominant diet, especially for the impact on gut microbiota composition, which varies significantly depending on VC presence. Magnesium has been shown to prevent VC but only in experimental and small clinical studies. Vitamin K has drawn considerable attention due to its activation of VC inhibitors. There are positive studies; unfortunately, recent trials failed to prove its efficacy in preventing VC. Future research is needed and should aim to transform food into a medical intervention to eliminate VC danger in CKD.

## 1. Introduction

Chronic kidney disease (CKD) is an emerging public health priority associated with high mortality rates and demanding complex management, including lifestyle changes, medications, and, sometimes, renal replacement therapy. Due to its very high prevalence in over 10% of the general population, CKD has a heavy social and financial burdens [1]. Above all other effects, CKD produces a significant negative impact on patients’ lives, leading to complications that affect their quality and becoming life-threatening over time. One of the most important, yet unsolved, complications is chronic kidney disease—mineral and bone disorder (CKD-MBD) [2,3,4]. As CKD progresses, cardiovascular and osteo-articular complications occur and may have different impacts on patients’ quality of life. CKD-MBD leads to a multitude of symptoms, including decreased function and social roles, depression, and a shorter life span [4,5]. Vascular calcification (VC) is the main abnormality from the complex CKD-MBD in terms of associated cardiovascular morbidity and mortality [2,3,6]. The goals of management in different stages of CKD are to slow the progression of kidney disease, to postpone the need for replacement therapy, and to control complications, such as hyperkalemia, metabolic acidosis, inflammation, protein malnutrition, anemia, high blood pressure, and mineral and bone disorders, which are particularly important for chronic management [1].

Food and drug intake may sometimes have a poisonous effect on the kidneys; therefore, a large number of patients with kidney failure have reached a point of no return due to their everyday life choices. Lifestyle impacts epigenetics, body composition, and function, so people will eventually develop CKD if their habits are harmful. In the most prominent diseases of this century, including diabetes mellitus, hypertension, cardiovascular diseases, and cancer, studies have been carried out and have proven that different environmental factors contribute to each of these diseases’ pathogeny [7,8]. Type 2 diabetes mellitus is associated with a diet that is rich in sweets, soft drinks, snacks, nuggets, and other ultra-processed foods [7]; hypertension has a strong connection with salt intake [9]; and cardiovascular diseases have strong connections with fatty foods. And, as is well known, the above-mentioned illnesses are the main causes of CKD. The ageing process itself is associated with common “burden of lifestyle” diseases, which include CKD. Interestingly, aging and CKD share important features; CKD is a condition which leads to an increased biological age [10], and CKD-MBD raises its invalidity rate and death toll [11]. We live in times of increasing awareness of the potential of meals to damage health. Consequently, a useful idea that has captured public attention is that food may become a tool to prevent or treat diseases and might be considered as medicine. This idea was conceptualized as medical nutritional therapy [12] or food as medicine [13,14]. When properly used, food may heal and may slow down and alleviate disease. Besides all of these well-known factors, different dietary patterns may be important influencers for chronic diseases, including CKD and its main chronic complication, CKD-MBD. Energy and action define everyone’s way of living; therefore, smart choices must be made regarding food as energy supply based on valid scientific data. There is hope that lifestyle changes will slow or stop CKD-MBD features and prevent or even lead to regression in VC [15,16,17]. Clear knowledge about what to eat, how much to eat, and in what combination is necessary for patients with CKD-MBD [15]. Medical nutrition therapy (MNT) is an evidence-based process aiming to treat or manage a disease through nutrition. Its components are comprehensive and include the evaluation of nutritional status, intervention in diets, and nutrition therapies [12].

In this study, we aim to provide a comprehensive review of the effects of diet on VC as a part of the CKD-MBD spectrum that is known to be associated with severe clinical outcomes in patients with CKD. We will present the current state of this research field by reviewing the key publications from recent years, and we will highlight controversial and diverging hypotheses regarding this approach.

## 2. Vascular Calcification in CKD

CKD is characterized by features of accelerated ageing, such as increased levels of cellular senescence, and epigenetic modifications, such as telomere attrition, arterial calcification, osteoporosis, sarcopenia, frailty, and depression [11]. According to the KDIGO guidelines, the term “chronic kidney disease—mineral and bone disorder (CKD–MBD)” is a clinical syndrome which comprises mineral, bone, and calcific cardiovascular abnormalities in CKD. It includes modifications of calcium, phosphorus, parathyroid hormone (PTH), vitamin D, bone metabolism, and vascular or other soft-tissue calcification [2,3]. CKD-MBD is a consequence of CKD that has led to extended research and the development of a wide variety of treatments; despite specialists’ implications, it continues to produce a multitude of symptoms and deleterious effects for the people who have it, including the following:

- Vascular calcification (VC) is a phenomenon involving the deposition of calcium and phosphorus within the layers of the arteries. Medial calcification, which presents as rail-train deposits along the vasculature, is particularly prevalent in patients suffering from CKD, but it is associated with aging and diabetes mellitus, too. It mainly affects the aorta and peripheral arteries. The deposition of mineral content within the media is preceded by phenotypic changes in vascular smooth muscle cells (VSMCs) and leads to arterial stiffness, significantly contributing to heart failure and increased cardiovascular morbidity. The accumulation of uremic toxins, the imbalance of calcium and phosphate, and a lack of calcification inhibitors have been implicated in the pathogenesis of calcification.
- Intimal calcification displays a patchy distribution pattern and preferentially affects the coronary and carotid arteries. It is part of the atherosclerosis process. In patients with dyslipidemia and hypertension and smokers, atherosclerotic plaques occur as a consequence of inflammation and endothelial damage. It is common to find both types of calcifications in CKD patients. Accumulation of mineral content in atherosclerotic plaques may increase the risk of ischemic events such as stroke, ischemic coronary syndromes, or ischemic arteriopathy of the lower limbs [18,19].
- Other ectopic extraskeletal calcifications may occur. Valvular calcification is highly prevalent in CKD patients, contributing to chronic heart failure; calcifications in the joints can cause pain and functional impotence, and calcifications in the subcutaneous tissue can lead to resistant pruritus.

## 3. Food for CKD Patients

According to Global Burden of Disease Study, dietary risk factors are major contributors to millions of deaths, leading to higher mortality rates than well-known risk factors such as smoking [20]. Lifestyle interventions, such as healthy nutritional habits, proved to be effective in reducing cardiovascular risk factors in the general population [21]. High intake of sodium and sugar and a low intake of whole grains, vegetables, and fruits can cause type 2 diabetes mellitus, hypertension, cardiovascular disease, cancer, and CKD [22]. A study conducted in the Netherlands on over 78,000 people with a follow-up of 3.6 years revealed new evidence that ultra-processed food consumption leads to kidney function decline [23]. An observational study from Brazil demonstrated that elderly patients on hemodialysis (HD) have a worse dietary quality and higher consumption of ultra-processed food than elderly without CKD [24]. Some diets, as the DASH (Dietary Approaches to Stop Hypertension) diet and Mediterranean diet, provided important evidence regarding efficacy in promoting health [25]; these diets especially involve the reduction in salt, fat, and processed food intake. Tyson et al. demonstrated in CKD patients that the reduced-sodium DASH diet is efficient in reducing blood pressure [26].

CKD people are constantly exposed to conditions that alter epigenetic regulation such as toxins and shifts in dietary patterns. CKD-MBD leads to changes in DNA or histones, which are heritable from one cell to its descendants [27]. Neytchev et al. compared dialysis patients with transplant patients and controls and demonstrated that the uremic milieu drives genome-wide methylation changes that are partially reversed with kidney failure replacement therapy [28]. Studies have shown that different life variables, including food choices, may lead to epigenomic reprogramming [27,29]. Recent research of these nutritional interventions in CKD patients with VC gives rise to the hope of finding solutions.

## 4. Phosphorus, Vitamin D, and Calcium and Vascular Calcification in CKD

In patients with CKD, mineral disorders are associated with hyperparathyroidism, renal osteodystrophy, arterial calcification, and cardiovascular mortality [30,31]. CKD-MBD is marked by high serum phosphate levels, low serum active vitamin D, and low serum calcium levels.

Increased phosphate levels lead to VC and high cardiovascular death. In their experimental study, Turner et al. discovered that the arteries acutely deposit large amounts of amorphous phosphate to control the elevation in the bloodstream, thereby altering the systemic disposition of phosphate; therefore, they identified the arteries as a participatory mineral homeostatic organ [32]. Nephrologists encounter serious difficulties in controlling phosphate levels, and phosphate impacts the CKD-MBD patients’ prognosis, even when receiving specific medication. Yet, the benefits of phosphate-lowering medication on VC, arterial stiffness, and clinical outcomes in predialysis CKD stages remain uncertain [33,34]. Nevertheless, there is evidence in favor of phosphate lowering; a recent Japanese study has proven that consistently strict phosphate control may slow the progression of coronary and valvular calcifications in incident patients undergoing HD (Table 1) [35]. 

In PROGREDIR study on non-dialysis CKD patients, Machado et al. demonstrated that an increased intake of phosphorus- and calcium-rich food is associated with coronary artery calcification (Table 1) [36].

Sources of phosphorus include meats, fish, poultry, dairy products, nuts, beans, and food additives. Animal foods and inorganic phosphorus from food additives and preservatives have higher phosphorus absorption than plant foods; the industrial use of phosphate in additives used for ultra-processed food is strongly linked to cardiorenal disease risk [59,60]. An increasing number of specialists recommend a more plant-based diet to control phosphate. Phosphate bioavailability is lower with a vegetarian diet compared to a diet based on animal protein or processed foods and beverages. Many foods that have traditionally been labeled high in phosphate (such as beans and nuts) may actually be acceptable because phosphate from these sources is only partially and slowly absorbed. The plant-derived phosphate found in unprocessed foods is in the form of phosphorus phytate, and the human intestine does not secrete phytase, the enzyme required for absorption [60,61]. In addition, such a diet, rich in legumes, nuts, and whole grains, may also result in higher fiber intake while offering wider food choices and preventing constipation with better digestive phosphorus elimination [62,63]. These data highlight the importance of phosphate bioavailability in different foods in CKD patients as a mediator of cardiovascular risk.

In cases of severe and progressive secondary hyperparathyroidism, the 2017 KDIGO guidelines recommend the use of calcitriol and vitamin D analogs [3]. We have to be aware of the potential double-edged sword effect of vitamin D, since both deficiency and excess may be related to VC. While deficiency produces hyperparathyroidism and VC, treatment with calcitriol and vitamin D analogs, even if reducing the PTH level, can lead to the development of VC by increasing the intestinal absorption of calcium and phosphate [64]. The intake of food rich in vitamin D, such as ergocalciferol and cholecalciferol, can decrease the required dose of active vitamin D, thus mitigating the VC risk associated with the latter. Vitamin D2 (ergocalciferol) and vitamin D3 (cholecalciferol) are recognized as fat-soluble prohormones, having different sources. Part of vitamin D as a nutrient is synthesized by the body through the action of sunlight, and some foods are fortified with the vitamin. Yet, there are foods naturally rich in vitamin D, including salmon, herring, mackerel, sardines, mushrooms, cashews, and hazelnuts [22,65]. Well controlled studies are needed to determine whether nutritional vitamin D slows the rate of progression of VC.

## 5. Magnesium and Vascular Calcification in CKD

The capacity of magnesium to inhibit calcium phosphate crystallization has been well documented in the context of VC. Magnesium effectively suppresses phosphate-induced calcification of VSMCs, as proved in different experiments [66]. Magnesium is known to suppress the maturation of calciprotein particles, which may play a pivotal role in the pathogenesis of VC. A high-magnesium diet prevented aortic calcification in animal models of CKD, such as Klotho knockout mice [37] (Table 1).

Patients with diabetes and CKD on HD showed reduced carotid intima–media thickness after magnesium supplementation, emphasizing a preventive role against VC [38]. Sakaguchi conducted a randomized trial comparing magnesium oxide and oral carbon adsorbent in predialysis CKD patients with coronary artery calcifications. The study proved the efficacy of magnesium to prevent coronary artery calcification progression [39] (Table 1).

A systematic review analyzed prospective clinical trials testing interventions to attenuate VC in people with CKD. It concluded that, in general, data are insufficient or conflicting, yet magnesium appears to be one of the few promising therapies [67].

The more recent Magical-CKD trial failed to demonstrate an improvement of coronary artery calcification progression after magnesium supplementation [40] (Table 1). Nevertheless, magnesium is one of the few nutritional elements with supportive data in terms of VC protection. Hypomagnesemia is not rare in patients with CKD, and several causes can be identified; the dietary restriction of potassium limits the intake of magnesium, and diuretics are known to enhance its urinary excretion. Almonds, peanuts, cashew, and spinach are foods rich in magnesium; these can be a good source especially in patients with low serum magnesium levels.

Zinc is considered an essential nutrient, having numerous benefits for health. A recent study demonstrated associations of low blood zinc levels with coronary artery calcification and future cardiovascular events in CKD patients. Good sources of zinc include seafood, meat, nuts, whole grains, and dairy products, which are recommended to avoid a zinc deficit [41].

## 6. Vitamin K and Vascular Calcification in CKD

**Figure 1:**
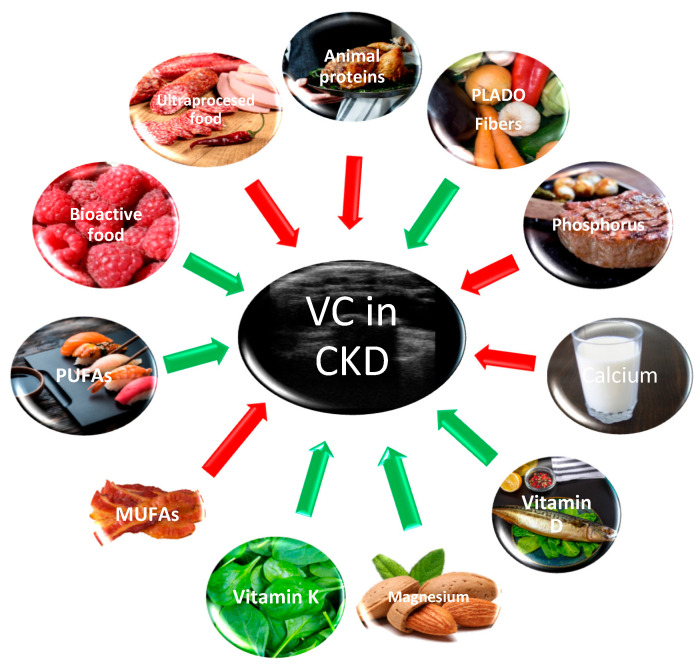
Food and vascular calcification (VC) in chronic kidney disease (CKD). Green arrows refer to a protective effect of the nutritional components against VC and red arrows indicate favorable VC development.

There is a close relationship between vitamin K and biomineralization. Vitamin K enables normal calcification processes in bones and soft tissues. This role is associated with vitamin-K-dependent proteins, including osteocalcin, matrix γ-carboxyglutamic acid (Gla) protein, and growth arrest specific 6 (Gas6). Matrix Gla protein, a vitamin K-dependent protein produced by VSMCs, is a powerful inhibitor of VC in culture cells with medial and intimal calcification. In view of the key role played by vitamin K, it is not surprising that patients with vitamin K deficiency and those who are using long-term anticoagulant therapy with vitamin K antagonists are prone to develop VC. There are two types of vitamin K. Vitamin K1 (phylloquinone) is found primarily in foods, especially plant-based oils, green vegetables (e.g., broccoli, spinach, and cabbage), and cow’s milk. The forms of vitamin K2 (menaquinones) are produced by bacteria, being found in meat, dairy products, and fermented foods, and are also synthesized in the intestine by colonic bacteria [68] (Figure 1).

McCabe et al. studied rats with adenine-induced chronic renal failure and showed that administration of a high dose of vitamin K protected against the development of warfarin-induced calcification [42] (Table 1).

In a study on HD patients, the levels of matrix Gla proteins displayed a significant increase in patients receiving vitamin K2 compared with vitamin K1 and placebo groups [43].

A study on HD patients from China demonstrated that the VC scores decreased as an effect of a vitamin-K-enriched dialysate [44].

Recently, a few randomized controlled trials were designed to test the anticalcification properties of vitamin K. Trevasc-HDK failed to prove that vitamin K2 can reduce progression of coronary artery calcification in HD patients [45]. The conclusion of RenaKvit, a double-blind, randomized, placebo-controlled trial, was that vitamin K supplementation does not modify the progression of arterial calcification in dialysis [46]. Similar results were reported by the iPACK-HD trial; there was improvement in vitamin K levels but no significant modification of VC progression [47] (Table 1).

In conclusion, vitamin K had no consistent benefit in VC reduction in CKD patients. Vitamin K1 showed better efficacy in correcting vitamin K status, and it had very positive results in experimental studies as a protector against VC. Further clinical studies are needed to shed light on the effect of vitamin K supplementation on arterial health, mostly because there is hope from experimental studies.

Regarding the effects of other vitamins, vitamin E has proven anti-atherogenic and antioxidant attributes, which have been correlated with improved cardiovascular outcomes. Wheat germ oil, sunflower seeds, and avocado have an increased content of vitamin E [69]. A recent study on non-dialysis CKD patients suggested that a higher intake of vitamin B5 (pantothenic acid) may have a small protective effect on coronary calcification [36].

## 7. Lipids and Vascular Calcification in CKD

Dyslipidemia plays a pivotal role in arterial intima calcification. Among the risk factors for atherosclerosis, cholesterol and lipid deposition are strongly associated with plaque formation and calcification. Clinical trials involving HMG-CoA reductase inhibitors showed good efficacy in reducing lipid levels and cardiovascular risk yet could not consistently demonstrate attenuation of VC [67]. KDOQI guidelines for nutrition in CKD highlight the importance of food choices and suggest that prescribing a Mediterranean diet may improve lipid profiles in adults with CKD 1–5 not on dialysis, having dyslipidemia or not. Prescribing increased fruit, legume, and vegetable intake may decrease body weight and blood pressure in CKD 1–4 patients [70].

Omega-3 polyunsaturated fatty acids (PUFAs), particularly eicosapentaenoic acid and docosahexaenoic acid, are part of a class of lipids with various biological functions. They reduce inflammation and atherogenesis, and, as a result, they can decrease the cardiovascular mortality [71]. Omega-3 PUFAs are used as medication for hypertriglyceridemia in patients with CKD. They are also a component of food, being present in fish oil; in a variety of microorganisms, including bacteria and marine microalgae; and in plant sources, such as flaxseeds, chia, and walnuts [72] (Figure 1). When it comes to mortality and cardiovascular disease, the current KDOQI guidelines in nutrition do not routinely recommend PUFA supplementation, even if it is acknowledged that lipid profile will be improved [70]. Several studies have investigated PUFAs’ effects in CKD; PUFAs increase the membrane potential and ATP levels in mitochondria with a protective effect on the kidneys and arteries. A recent study demonstrated that maslinic acid can reduce renal interstitial fibrosis and can prevent CKD progression and complications [73]. Interestingly, randomized controlled trials comparing the effect of omega-3 PUFA supplementation with placebo have shown significant relief of uremic pruritus, which is associated with CKD-MBD [74].

Experimental studies documented a preventive role of omega-3 fatty acids in pathological calcification, leading to decreased warfarin-induced medial arterial calcification in a rat model [48] (Table 1). It has been reported that patients with CKD have low serum expression of Klotho, which has been proved to be an arterial calcification inhibitor [75]. Nakamura et al. has shown that eicosapentaenoic acid can limit arterial calcification in Klotho mutant mice [49] (Table 1). A recent large study demonstrated an association of higher plasma levels of omega-3 PUFAs with an increased arterial elasticity [76]. 

Monounsaturated fatty acids are known to have deleterious effects on health (Figure 1). As opposed to PUFAs’ protective attributes, Son et al. demonstrated that the erythrocyte membrane content of monounsaturated fatty acids is significantly higher in HD patients with arterial medial calcification of the feet than in the patients without calcifications [50].

The ketogenic diet, which consists of a limited intake of carbohydrates and a liberal intake of fats, has recently attracted considerable interest. It is proven as an efficient intervention in controlling type 2 diabetes mellitus [77] and in slowing renal cyst growth [78]; therefore, patients with diabetic kidney disease and autosomal-dominant polycystic kidney disease may derive benefits from this diet, including a delay in progression and, eventually, in the complications rate [79]. It may cause a rise in cholesterol levels, so, when adopted, it should be accompanied by close monitoring and treatment for dyslipidemia [77]. Dietary modulation can increase and maintain circulating ketone bodies, especially β-hydroxybutyrate (β-HB), which is one of the most abundant ketone bodies in human circulation [80]. A very important finding was reported by Lan et al.; the ketone body β-hydroxybutyrate (BHB) produced in the ketogenic diet has been demonstrated to suppress VC in CKD through downregulation of HDAC9 [51] (Table 1).

Intermittent fasting, as a model of caloric restriction, has broad-spectrum benefits for many health conditions, such as atherosclerosis, cardiovascular disease, and obesity, as preclinical studies and clinical trials have shown [81]; as far as the impact on VC, it remains a topic for future studies.

## 8. Uremic Toxins, Microbiota, Fibers, and Vascular Calcification in CKD

Accumulation of various uremic toxins, including inorganic phosphate (Pi), interleukins (IL-1β, IL-6), tumor necrosis factor alpha (TNFα), and indoxyl-sulfate, have been linked to VC. Pi induces the upregulation of several osteoblast-like transition molecules like BMP2 (bone morphogenetic protein 2), RUNX2 (Runt-related transcription factor 2), and osteopontin that initiate the pro-calcifying trans-differentiation of VSMCs. Indoxyl-sulfate stimulates transforming growth factor beta (TGFβ) expression and medial layer hyperplasia. Uremic toxins act on endothelial cells to induce vasoconstriction, upregulation of extracellular matrix degradation molecules such as matrix metalloproteinases 2 and 9, and oxidative stress. Calcium and Pi deposition in the form of hydroxyapatite crystals induces medial VC [82].

The load of uremic toxins can be reduced through dialysis, yet their production is influenced by dietary habits, mostly important in pre-dialysis stages. Some foods are sources for protein-bound uremic toxins (e.g., indoxyl sulphate). These are by-products of aromatic amino acids (phenylalanine, tryptophan, and tyrosine) from protein disintegration by gut microbiota [83]. Colonic bacteria transform tryptophan to indol, which, through oxidation and sulfation in the liver, will lead to indoxyl sulphate formation. Rodrigues et al. have similarly explained the pathophysiology of the interplay between gut microbiota, bone health, and VC in CKD [84]. Interesting results come from studies investigating the influence of different dietary habits on the uremic gut microbiota. Merino-Ribas et al. found differences in the type of microbiota of CKD on peritoneal dialysis with or without VC, namely Coprobacter, Coprococcus 3, Lactobacillus, and the Eubacterium eligens group in the gut and Cutibacterium, Pajaroellobacter, Devosia, Hyphomicrobium, and Pelomonas in the blood. These results may indicate a link between microbiota and VC in CKD patients on peritoneal dialysis [52] (Table 1). Such results from similar studies led to the hypothesis that inflammation and gut dysbiosis are important drivers of CKD–MBD [85]. An association between dietary inflammatory index and cardiovascular disease and mortality was recently proven [86]. A study identified an association of the proatherogenic metabolite trimethylamine N-oxide (TMAO), which increases due to gut dysbiosis, with cardiovascular outcomes in HD patients [87].

Lactobacillus rhamnosus GG is a probiotic with great promise in bone formation, but an experimental study recently proved an association with worsening of VC in CKD [53] (Table 1).

Fiber intake is an important health promoter in the general population. In their recent study, conducted on over 3800 Korean patients with CKD, Kwon et al. reported an inverse association between dietary fiber intake and all-cause mortality at 10 years in CKD patients [88]. Higher fiber intake was associated with less inflammation, less myocardial hypertrophy, and lower risk of cardiovascular events in dialysis patients [89]. Fibers are needed for the effective absorption of nutrients. Fibers demonstrated salutary benefits, including improved glycemic and lipid control, blood pressure, gastrointestinal motility, and gut microbiota composition [90]. In a study published on HD patients, increased dietary fiber intake led to the reduction in indoxyl sulphate levels by 29% [91].

Adequate consumption of phytate (containing myo-inositol hexaphosphate) can prevent abdominal aortic calcification in patients with CKD [54]. The phytate comes from whole-grain cereals, bran, and lentils. Nuts are also a good source of antioxidants and dietary fiber (Figure 1). The Calipso trial demonstrated the effect of myo-inositol hexaphosphate in slowing progression of cardiovascular calcification in patients on HD [55] as additional evidence for the usefulness of this component of fibers (Table 1).

Finding the balance in gut microbiota and regulating microbiota-derived metabolites by dietary intervention and probiotics are new targets for the improvement of the gut–kidney–arteries axis, which indicates innovative interventions of VC in CKD [16,92].

## 9. Protein Intake and Vascular Calcification in CKD

The foundation of nutrition intervention in CKD was laid for decades on a low-protein diet to slow progression and on restriction of plant foods, such as vegetables and fruits, to prevent hyperkalemia. Lately, this paradigm has changed, and the plant-dominant low-protein (PLADO) diets seem to have become a better choice for patients with CKD [21]. In a sub-analysis of the NHANES III study on 14,000 participants patients with a glomerular filtration rate < 60 mL/min, a diet with a higher proportion of protein from plant sources was associated with lower mortality, probably due to lower production of uremic toxins and lower serum phosphorus levels [30]. The vegetarian diet or a reduced intake of red meat has been associated with a reduction in the generation of uremic toxins [93,94,95,96]. Such a diet is based on fruits, vegetables, seeds, nuts, tea, cocoa, and whole-grain cereals [97]. Among plant-based foods, Brazil nuts seem to have important benefits in CKD, even in end-stage kidney disease patients, due to their contents of proteins, selenium, omega-3 fatty acids, and fibers [98,99] (Figure 1).

The DIET-HD study demonstrated for a large number on HD patients that the highest intake of fruit and vegetables had the lowest risk for all-cause and cardiovascular mortality [100,101]. On the contrary, the CRIC study did not find a significant association between higher diet scores and reduced risk for atherosclerosis or mortality [102] (Figure 1).

As for the risk of hyperkalemia, CKD patients have been advised for a long time to reduce their intake of fruits, vegetables, and nuts. Nevertheless, we must be aware that meat and ultra-processed food have a high content in potassium, with a high absorption rate [103,104,105,106,107,108,109].

Nuts have high content of phosphorus, which is one of the traditional nutrients restricted in advanced CKD to avoid hyperphosphatemia [108], but the latest studies demonstrated that non-animal protein does not lead to hyperphosphatemia, as previously believed [105,109].

To identify dietary components associated with abdominal aorta calcification, data from NHANES were employed in a cross-sectional study. Low contents of proteins, fiber and vitamin A, and high contents of lipids and caffeine exhibited an association with abdominal aorta calcification. High adherence to the plant-based pattern was associated with a lower risk of VC, as a new and valuable result in favor of PLADO [56].

## 10. Bioactive and Senolytic Food and Vascular Calcification in CKD

Bioactive and senolytic food has antioxidant and anti-inflammatory effects. Resveratrol, quercetin, curcumin, anthocyanins, and cruciferous and cocoa powder are part of this category [110] (Table 1). Anthocyanins, present in purple fruits and vegetables, exert their beneficial effects through improvements in oxidative stress, inflammation, gut microbiota, and modulation of neuropeptides. Their health benefits in humans include protection of the cardiovascular system and kidneys, among others [111].

Resveratrol, a dietary polyphenol compound, has anti-inflammatory and antioxidative properties [112]. Recently, studies also showed that resveratrol is a scavenger for many free radicals and ameliorates VC in CKD [57].

Cocoa contains fatty acids and polyphenolic bioactives, with proanthocyanidins being the most abundant and methylxanthine alkaloids. Dark chocolate led to a reduction in TNFα and no change in potassium and phosphorus plasma levels. These are the results of a clinical trial of 2 months on HD patients [113] (Table 1).

Blueberry, cranberry, raspberry, and strawberry are modulators of the gut microbiota and a target for treatment of gut dysbiosis in CKD [114] (Figure 1).

Dietary senolytics, such as quercetin (found in apples), fisetin (in strawberries), and organosulphur compounds and flavonoids (aged garlic) may be alternative approaches to reduce cardiovascular risk in CKD [13,115]. Quercetin exerted a protective effect on VC in adenine-induced chronic renal failure rats, possibly through the modulation of oxidative stress [58] (Table 1).

Iron supplementation is highly recommended to improve cardiovascular function in CKD, but it remains controversial when it comes to VC. Recent studies demonstrated that iron targets some pathways of VC dependent on phosphorus-induced osteoblastic transformation of VSMCs to calciproteins, apoptosis, and inflammation, since it is effective both in prevention and when calcification is already established [116].

Selenium works as an antioxidant in the body by preventing vascular cell damage. In a recent study, a higher dietary selenium intake was negatively associated with severe abdominal aorta calcification incidence in CKD patients [117]. The selenium content of foods can vary considerably depending on the geographic area; nuts, oats, seeds, mushrooms, beans, and eggs can be good sources.

As for all the benefits discovered in the mentioned studies, the concept of food as medicine for protecting the kidneys and heart and avoiding VC in CKD patients seems to have moved closer to reality [118].

## 11. Conclusions

The search for eternal youth, as an emblem for health, is as old as mankind. But in the case of the patients with CKD and VC, it is more of a struggle for life, a fight against many deadly factors, because VC is strongly associated with cardiovascular mortality. Most of the efforts are made to fix problems with a focus on the other end of the spectrum of CKD, and yet medication failed to show consistent efficacy in preventing VC.

Food is essential for life; thus, prevention of VC in CKD through nutrition seems to be the logical approach. High phosphorus absorption, high production of uremic toxins, and gut dysbiosis are consequences of the increased intake of animal-based proteins, processed food, salt, and sugar. Available research links all of the above with the presence of VC. A diet involving vitamin K, magnesium, plant-based diets, fibers, omega 3 fatty acids, or bioactive food appears to be the most promising in protecting against VC. These results are based on experimental and relatively small clinical studies but still are not negligible. Even though clinical trials on magnesium and vitamin K were not able to prove the efficacy of the nutritional interventions in CKD patients with VC, the effects exist, and more research needs to be conducted. Finding the best variants of meals may lead to reduced VC incidence and progression and may allow eating to be transformed into a scientific act and medical intervention with effective outcomes. Food is supplied for life, and data are available to be discovered on the best nutrient choices to disrupt the vicious cycle of the gut–kidney–arteries axis and prevent cardiovascular calcification in the CKD population.

## Figures and Tables

**Table 1:** The effects of food on vascular calcification in patients with CKD.

Article	Design	Results
Shimizu 2023 [35]	Japanese study 64 incident HD patients Phosphate levels CAC by CT scans	Consistently strict phosphate control may slow the progression of coronary and valvular calcifications
Machado 2018 [36]	PROGREDIR study 373 non-dialysis CKD patients Food questionnaire Coronary artery calcification (CAC) by CT scans	Increased intake of food rich in phosphorus, calcium, and magnesium is associated with CAC
Ter Braake 2020 [37]	Klotho-deficient mice High dietary Mg	Mg prevents VC in Klotho deficiency
Talari 2019 [38]	RCT 54 HD patients with diabetes Mg oxide or placebo	Decrease in intima–media thickness after Mg supplementation
Sakaguchi 2019 [39]	RCT of 96 non-dialysis CKD patients Mg oxide versus carbon adsorbent CAC by CT scans Follow-up 2 years	CAC score was significantly smaller in the Mg oxide group
Bressendorf 2023 [40]	Magical-CKD study 150 CKD patients Supplementation with Mg for 12 months CAC by CT scans	No effect on CAC
Zhang 2023 [41]	170 CKD patients and 62 healthy controls Blood zinc levels CAC by CT scans	Low zinc with moderate–severe CAC and CDV events
McCabe 2013 [42]	Rats with adenine-induced chronic renal failure and warfarin-induced VC	High dietary vitamin K1 increased vitamin K tissue concentrations and blunted the development of VC
El Shinnawy 2022 [43]	RCT on 120 HD patients given supplements of vitamin K2, vitamin K1, and placebo Matrix Gla protein levels	Matrix Gla protein levels showed a significant increase in the vitamin K2 group compared with vitamin K1 and placebo groups
Li 2017 [44]	100 HD patients Used vitamin-K-enriched dialysate	Decreased VC scores as the effect of vitamin K
Haroon 2023 [45]	Trevasc-HDK RCT on 138 HD patients; CAC scores Vitamin K2 supplementation	No effect on VC
Levy-Schousboe 2021 [46]	RenaKvit RCT on 48 dialysis patients Vitamin K or placebo for 2 years Abdominal aortic calcification	No difference in VC
Holden 2022 [47]	iPACK-HD RCT on 86 HD patients Vitamin K1 for 12 months Coronary artery calcium score	No difference in progression of coronary artery calcification
Kanai 2011 [48]	Warfarin-induced medial arterial calcification in a rat model	Decreased medial arterial calcification after omega-3 fatty acid supplementation
Nakamura 2017 [49]	Eicosapentaenoic acid in Klotho mutant mice	Eicosapentaenoic acid limit VC
Son 2012 [50]	Cross-sectional study 31 HD patients Erythrocyte membrane content of monounsaturated fatty acids Plain radiographs for VC	Monounsaturated fatty acid erythrocyte content is significantly higher in HD patients with arterial medial calcification of the feet than in those without calcifications
Lan 2022 [51]	Cell culture Animal studies Ketone body β-hydroxybutyrate and VC in CKD model	Ketogenic diet through β-hydroxybutyrate suppresses VC in CKD through downregulation of HDAC9
Merino-Ribas 2022 [52]	Cross-sectional study 44 CKD patients on peritoneal dialysis (PD) Gut and blood microbiomes VC on plain radiographs	Differences in microbiota between PD patients with and without VC
Wei 2023 [53]	CKD Rats with 1,25-dihydroxyvitamin D3 induced VC. Lactobacillus rhamnosus	Lactobacillus rhamnosus GG supplements worsened the VC in CKD
Sanchis 2016 [54]	69 non-dialysis CKD patients Food questionnaire evaluated the phytate (Myo-inositol hexaphosphate) intake. VC on plain radiographs	Increased phytate intake was associated with less abdominal aorta calcification
Raggi 2020 [55]	RCT 274 HD patients Myo-inositol hexaphosphate Cardiovascular calcification on CT scan 52 weeks	Slowed progression of cardiovascular calcification with myo-inositol hexaphosphate
Li 2023 [56]	Data from NHANES 1862 participants Information on 35 dietary components VC on plain radiographs	Low contents of proteins, fiber and vitamin A and high contents of lipids and caffeine were associated with abdominal aorta calcification. High adherence to the plant-based pattern was associated with a lower risk of VC
Zhang 2016 [57]	Resveratrol	Resveratrol is a scavenger for many free radicals and ameliorates VC in CKD
Chang 2017 [58]	Rats with adenin-induced chronic renal failure. Quercetin	Quercetin exerted a protective effect on VC

Abbreviations: CKD, chronic kidney disease; CAC, coronary artery calcification; HD, hemodialysis; Mg, magnesium; VC, vascular calcification; RCT, randomized control trial; HDAC9, histone deacetylase 9; NHANES, National Health and Nutrition Examination Survey.

## Data Availability

Data are contained within the article.
